# Supplementary material for: Electromagnetic polarization-controlled perfect switching effect with high-refractive-index dimers and the beam-splitter configuration
Source: Nat Commun. 2017 Jan 4;8:13910. doi: 10.1038/ncomms13910 (PMC5216122; doi:10.1038/ncomms13910)
Supplement: Supplementary Information — Supplementary Figures, Supplementary Notes, Supplementary Methods and Supplementary References [file ncomms13910-s1.pdf]

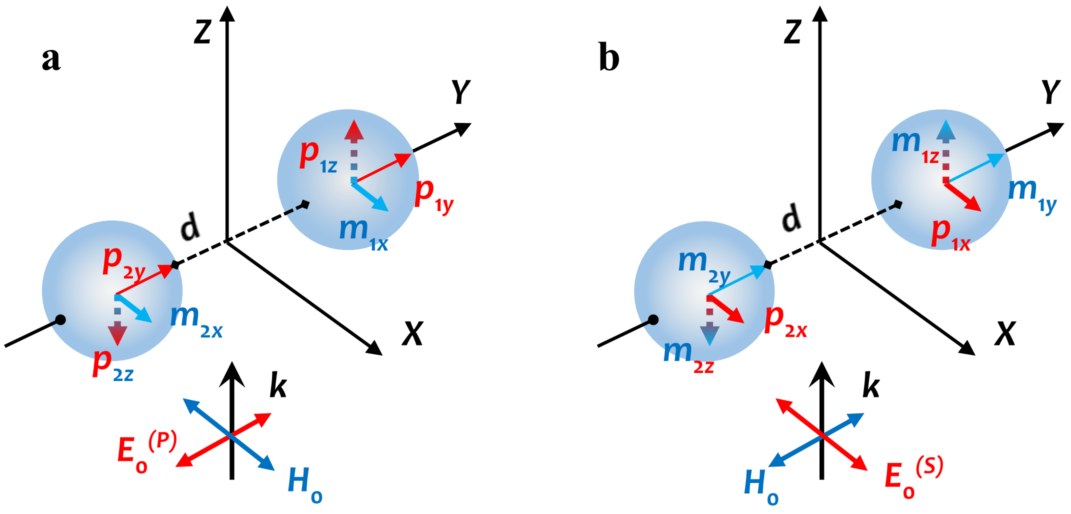

Supplementary Figure 1 | Orientation of the induced dipoles. a) Longitudinal configuration. **b)** Transverse configuration. The red vectors are related to the electric quantities, while the blue vectors are related to the magnetic ones. $\boldsymbol{E}_0$ corresponds to the polarization of the electrical incident field. $\boldsymbol{H}_0$ corresponds to the polarization of the magnetic incident field. Both correspond to a plane wave with incidence direction $\boldsymbol{k}$ ($|\boldsymbol{k}| = k = 2\pi/\lambda$ where λ is the incident wavelength). They generate an electric dipole $\boldsymbol{p}$ and a magnetic dipole $\boldsymbol{m}$ in each sphere. The interaction between the two spheres (each of radius R) generates a non-null Z-component for the electric dipole or for the magnetic dipole. This depends on both the orientation of the dimers and the polarization of the impinging wave. d corresponds to the gap distance between the two spheres.

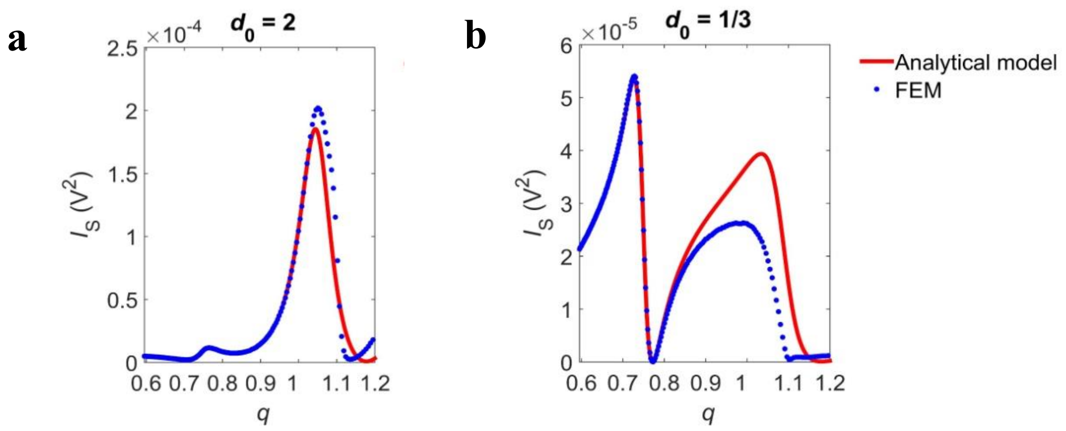

Supplementary Figure 2 | Validation of the dipolar approximation. Comparison of the scattering intensity I_S computed with the Finite Element method (FEM)[2] and with the dipolar approximation (Analytical model: Supplementary Equations (37-42)), for two gap values: **a)** d_0=2, **b)** d_0=1/3. $q=kR$ corresponds to the dimensionless size parameter and $d_0=d/R$ is the relative gap distance between particles, where R is the particle radius.

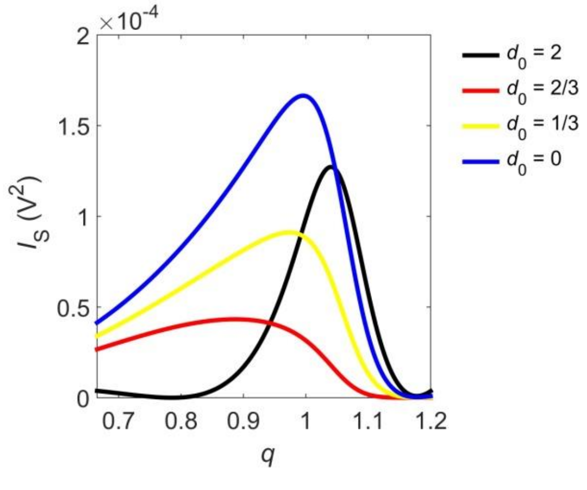

Supplementary Figure 3 | Influence of the interaction terms. Spectral behaviour of I_S when the interaction terms are neglected ($g_{zx}=g_{xx}=0$) in Supplementary Equations (31-34). Several gap distances d_0 are considered.

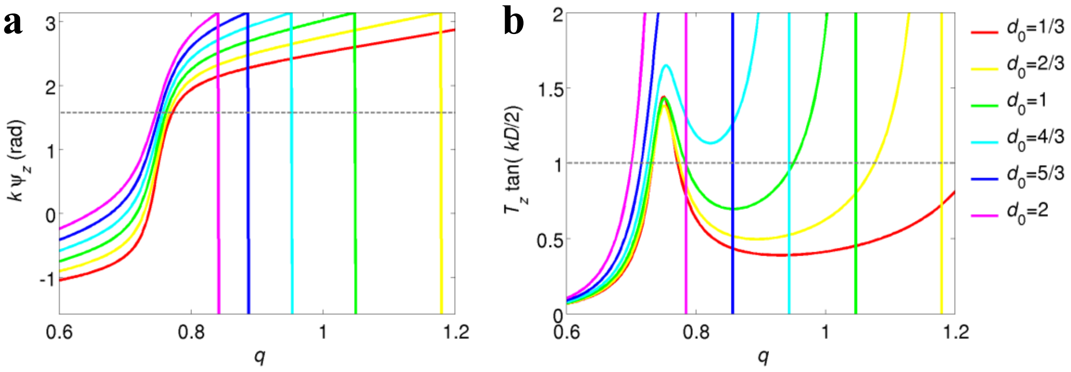

Supplementary Figure 4 | Evolution of the ratio between the *Z*-component of the induced magnetic dipole and the induced electric dipole. **a)** The phase difference between the two dipoles is plotted as a function of q. The line corresponding to $\pi/2$ has been plotted in order to detect the values of d_0 which lead to a minimum value of I_S, as indicated by Supplementary Equation (51). **b)** The amplitude ratio between the two dipoles is plotted as a function of q. The line corresponding to 1 has been plotted in order to detect the values of d_0 which satisfy Supplementary Equation (52). Several gap distances d_0 are considered.

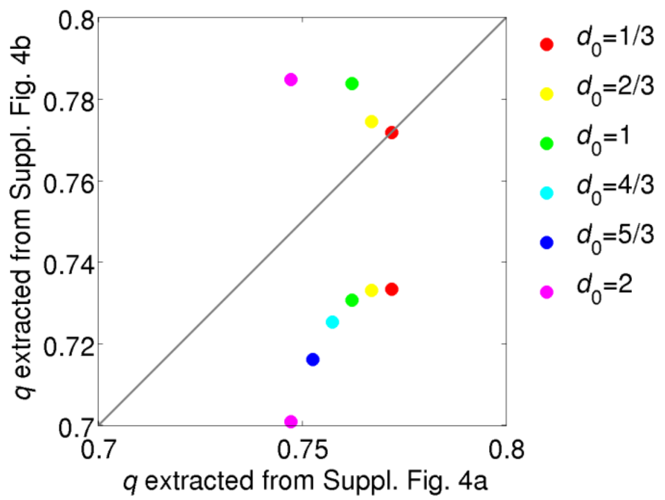

Supplementary Figure 5 | Search of the best gap satisfying the two constraints for T_z and ψ_z. For each gap, the value of the size parameter q which satisfies Supplementary Equation (51) has been manually picked from Supplementary Fig. 4a, as it corresponds to the intersection between the dotted line $\pi/2$ and the curve $k\psi_z$. This value of q is reported in abscissa. For each gap, the value of q which satisfies Supplementary Equation (52) has been manually picked from Supplementary Fig. 4b, as it corresponds to the intersection between the dotted line and the curve T_z tan(kD/2). This value of q is reported in ordinate. If the corresponding point is the closest from the identity line (shown in solid grey), it means that it best matches the two conditions of Supplementary Equations (51, 52). There are two separate sets of values as, in Supplementary Fig. 4b, the curve T_z tan(kD/2) intersects several times the dotted line in the interval range [0.7, 0.8].

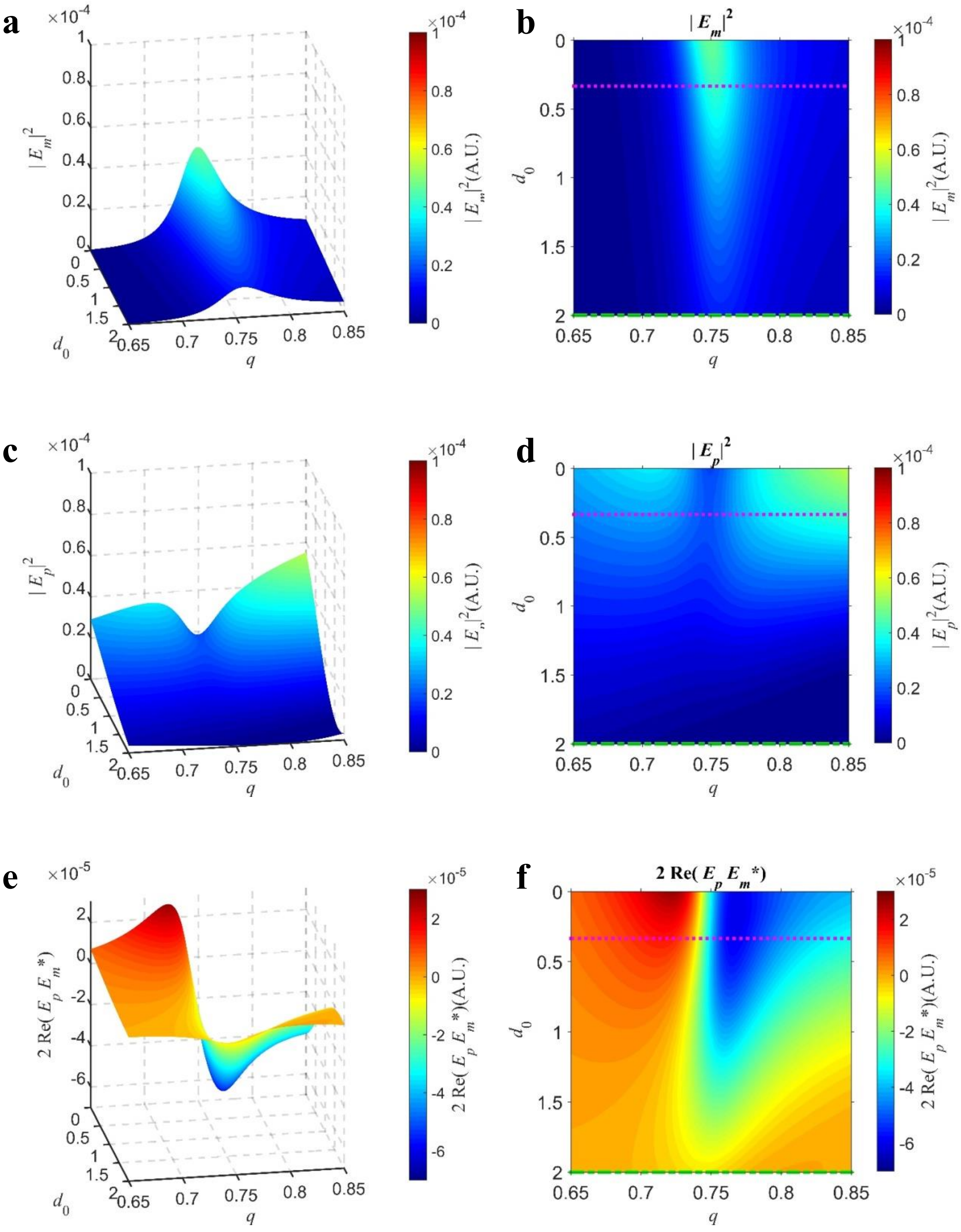

Supplementary Figure 6 | Terms of Supplementary Equation (53). Electric field amplitude created by the induced magnetic **(a-b)** and electric dipoles **(c-d)** in both particles and interferential term **(e-f)** in 3D and 2D respectively, as a function of the distance between the components of the dimer, d_0. The pink and green horizontal lines in **(b, d, f)** correspond to the strong and weak interaction configurations respectively, that means d_0 = 1/3 and d_0 = 2.

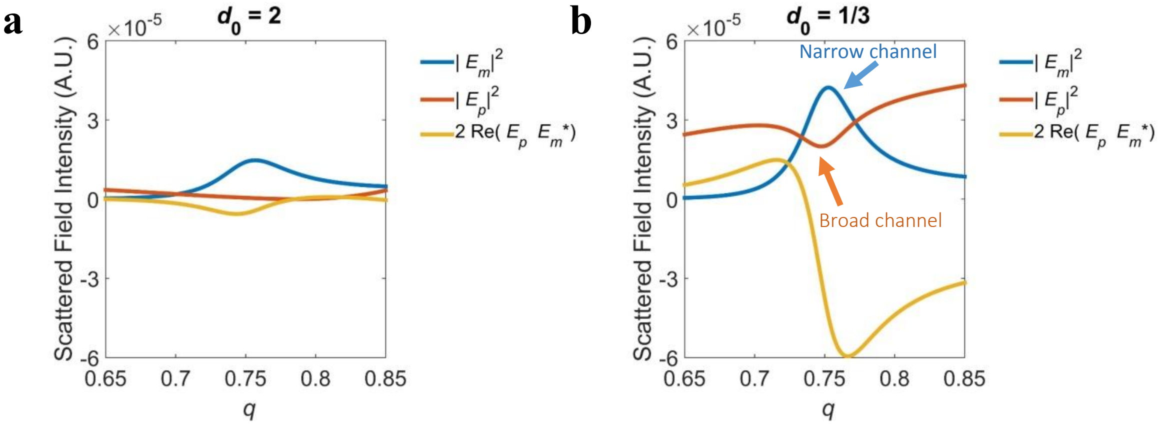

Supplementary Figure 7 | Terms of Supplementary Equation (53) for weak and strong interaction configurations. Electric field amplitude created by the induced magnetic and electric dipoles in both particles and interferential term for weak and strong interaction configurations: **a)** d_0 = 2 and **b)** d_0 = 1/3, respectively.

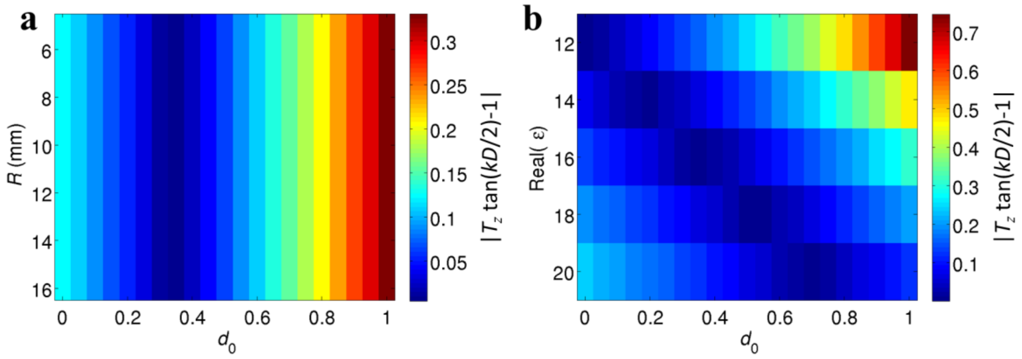

Supplementary Figure 8 | Search of the best gap satisfying the two constraints. For each gap d_0, the value of the size parameter q which satisfies Supplementary Equation (51) has been manually picked from Supplementary Fig. 4a. The associated value of T_z at that particular frequency is then computed and compared to 1, as indicated in Supplementary Equation (52). The color scale is thus directly showing the value of $|T_z \tan(kD/2)\text{-}1|$, which is a dimensionless quantity. **a)** The permittivity of the sphere is fixed to ε = 15.7+ 0.3i and the sphere radius changes. **b)** The radius of the sphere is fixed to R = 9 mm and the real part of the permittivity changes while its imaginary part remains fixed and equal to 0.3.

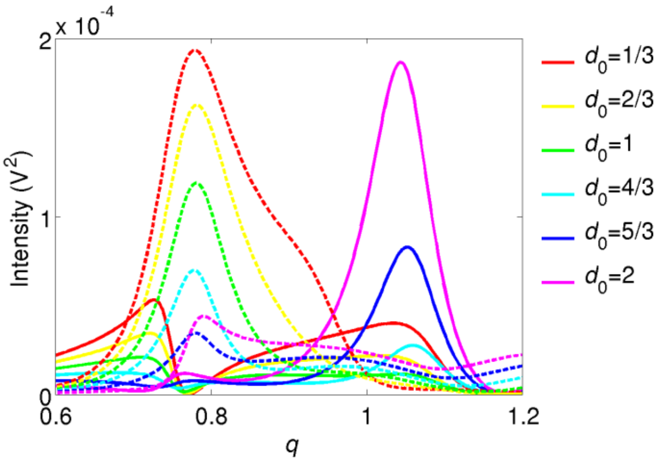

Supplementary Figure 9 | Spectral evolution of the intensities with respect to the distance between the two spheres. Spectral behaviour of I_S (solid lines) and I_P (dashed lines) plotted with respect to the size parameter q, when several gaps are considered. The gap d_0 =1/3 is responsible for a minimum value of I_S and a maximum value of I_P in the considered spectral range.

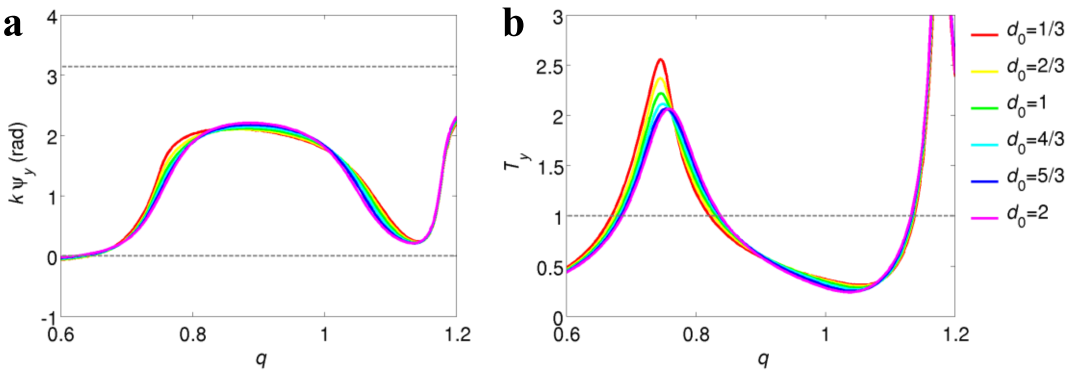

Supplementary Figure 10 | Evolution of the ratio between the *Y*-component of the induced magnetic dipole and the induced electric dipole. **a)** The phase difference between the two dipoles is plotted. The lines corresponding to *0* and π have been plotted in order to detect the values of d_0 which lead to a minimum value of I_S, as indicated by Supplementary Equations (64) or (65). **b)** The amplitude ratio between the two dipoles is plotted. The line corresponding to 1 has been plotted in order to detect the values of d_0 which satisfy the equal amplitude condition. Several gap distances are considered.

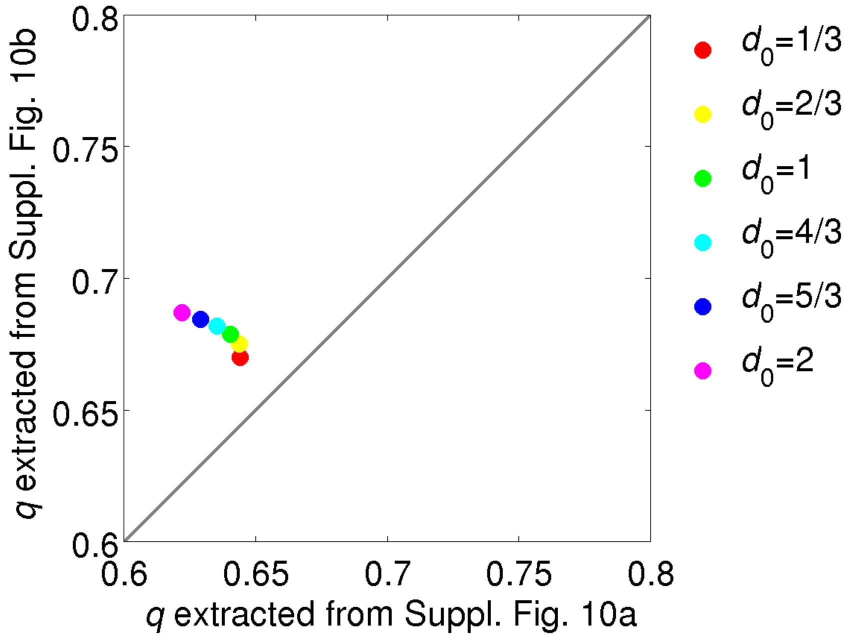

Supplementary Figure 11 | Search of the best gap satisfying the two constraints for T_y and ψ_y. For each gap, the value of the size parameter q which satisfies Supplementary Equation (66) has been manually picked from Supplementary Fig. 10a, as it corresponds to the intersection between the dotted line 0 and the curve $k\psi_y$. This value of q is reported in abscissa. For each gap, the value of q which satisfies Supplementary Equation (64) has been manually picked from Supplementary Fig. 10b, as it corresponds to the intersection between the dotted line and the curve T_y. This value of q is reported in ordinate. If the corresponding point is the closest from the identity line (shown in solid grey), it means that it best matches the two conditions (Supplementary Equations (64) and (66)).

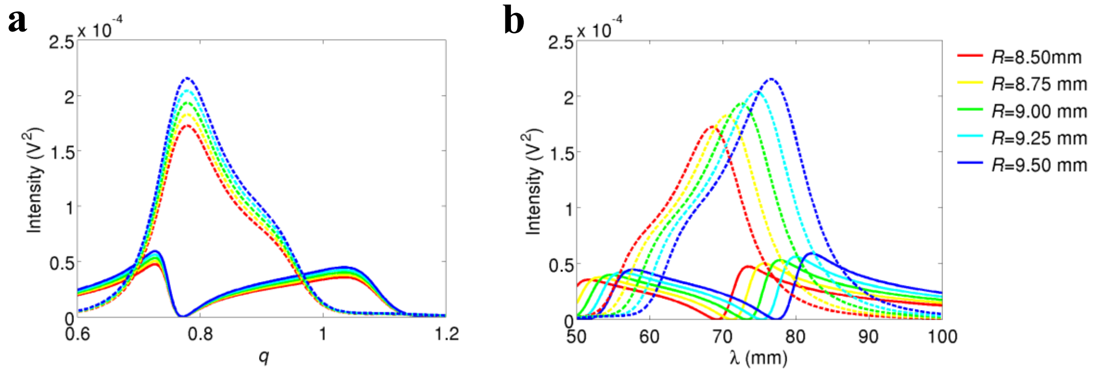

Supplementary Figure 12 | Spectral evolution of the intensities with respect to the radii of the spheres, *R*. Spectral behaviour of I_S (solid lines) and I_P (dashed lines) with respect to the size parameter q, when several sphere radii, R, are considered. A constant gap d_0=1/3 is kept in each case. The intensities are plotted either **a)** along the dimensionless parameter q or **b)** along the wavelength λ. All the other parameters are kept fixed ($\boldsymbol{k}=k\boldsymbol{e}_z$, θ=90°, ε=16.5 + 0.3i).

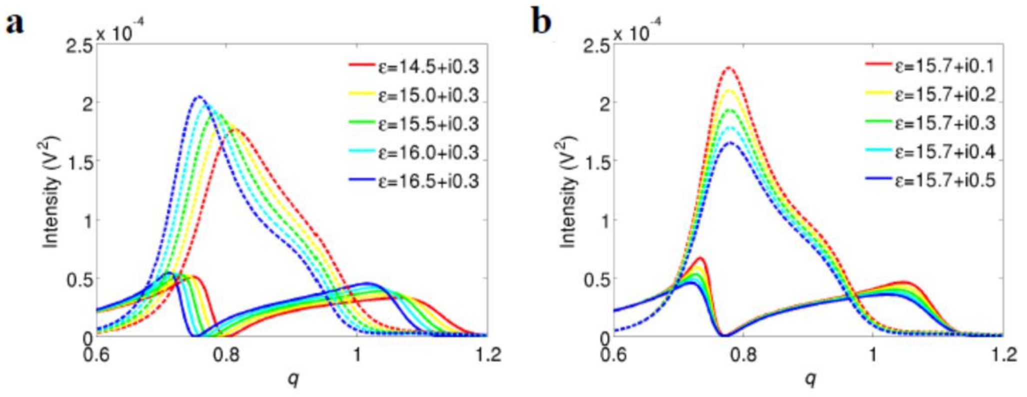

Supplementary Figure 13 | Spectral evolution of the intensities with respect to the index of the spheres. Spectral behaviour of I_S (solid lines) and I_P (dashed lines) with respect to the size parameter q, when several sphere indices are considered. A constant gap d_0=1/3 is kept in each case. **a)** The real part of the relative permittivity of the spheres varies while the imaginary part remains constant. **b)** The imaginary part of the relative permittivity of the spheres varies while the real part remains constant. All the other parameters are kept fixed ($\boldsymbol{k}=k\boldsymbol{e}_z$, θ=90°, R=9 mm).

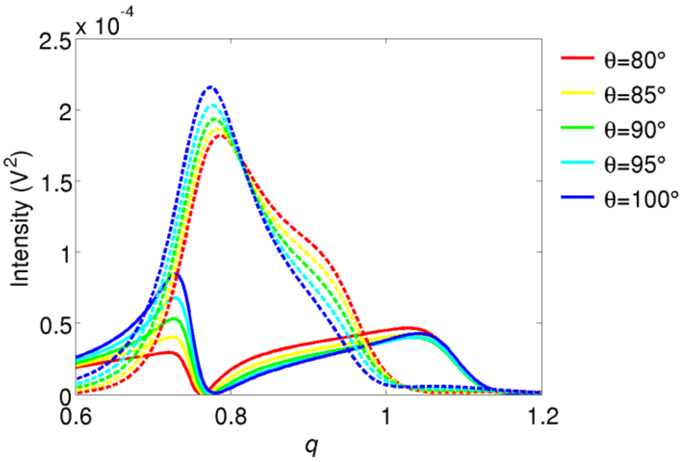

Supplementary Figure 14 | Spectral evolution of the intensities with respect to the receiving direction. Spectral behaviour of I_S (solid lines) and I_P (dashed lines) with respect to the size parameter q, when several sphere indices are considered. A constant gap d_0=1/3 is kept in each case. The receiver is positioned at 90° ± 10° from the spheres. All the other parameters are kept fixed ($\boldsymbol{k}=k\boldsymbol{e}_z$, R=9 mm, ε=16.5 +0.3i).

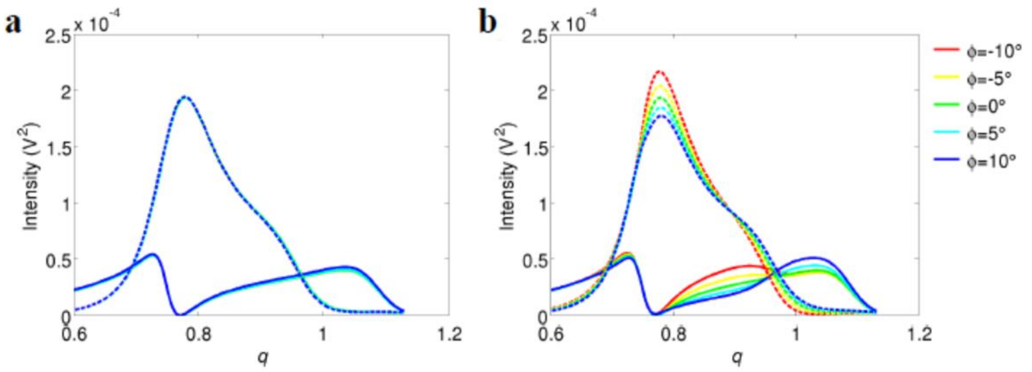

Supplementary Figure 15 | Spectral evolution of the intensities with respect to the alignment of the spheres. Spectral behaviour of I_S (solid lines) and I_P (dashed lines) with respect to the size parameter q, when several sphere positions are considered. A constant gap d_0=1/3 is kept in each case. **a)** The spheres are rotated in the *XY* plane along the *Z* axis with a variation of ±10°. **b)** The spheres are rotated in the *YZ* plane along the *X* axis with a variation of ±10°. All the other parameters are kept fixed (θ=90°, R=9 mm, ε=16.5 + 0.3i).

Supplementary Note 1

Green's method applied to the electric-magnetic dipole interaction. In this section, in order to explain the null values observed for I_S in Fig. 2, we analytically study the different contributions of both particles to the scattered intensity. In a similar way to the analysis performed in [1] for the longitudinal (I_P) configuration, here we analyze by means of the Green's function the interaction effect for the transverse (I_S) configuration. Fig. 1 depicts the same configuration as the one described in [1], with the polarization in the longitudinal (transverse) configuration along the Y-axis (X-axis).

In the transverse configuration case, as the impinging beam is assimilated to be a plane wave, the incident electric and magnetic fields are given by

$$\boldsymbol{E}_0(\boldsymbol{r}) = E_0 e^{ikz} \boldsymbol{e_x}, \tag{1}$$

$$\boldsymbol{H}_0(\boldsymbol{r}) = \frac{E_0}{Z} e^{ikz} \boldsymbol{e_y}, \tag{2}$$

where $Z=(\mu_0\mu_h/(\varepsilon_0\varepsilon_h))^{1/2}$ is the surrounding medium impedance, $k=(\varepsilon_h\mu_h)^{1/2}\omega/c$ is the wavenumber of the impinging radiation in the embedding medium with associated wavelength λ, $c=1/(\varepsilon_0\ \mu_0)^{1/2}$ is the speed of light in vacuum. ε_0 and μ_0 are the dielectric permittivity and magnetic permeability of the vacuum while ε_h and μ_h are the relative dielectric permittivity and magnetic permeability of the embedding medium.

Because the behaviour is mainly observed in the dipolar region, each one of the particles is modeled by two orthogonal dipoles, one electric $\boldsymbol{p}$ and one magnetic $\boldsymbol{m}$, which are both perpendicular to the propagation direction. The electric and magnetic scattered fields created by both particles of the dimer can be expressed attending to the Green methodology as

$$\boldsymbol{E}_{\mathrm{sca}}(\boldsymbol{r}) = \sum_{j=1,2}\left\{\frac{k^2}{\varepsilon_0\varepsilon_h}\boldsymbol{G}_{\mathrm{E}}(\boldsymbol{r}-\boldsymbol{r}_j).\boldsymbol{p}_j + iZk^2\boldsymbol{G}_{\mathrm{M}}(\boldsymbol{r}-\boldsymbol{r}_j).\boldsymbol{m}_j\right\}, \quad (3)$$

$$\boldsymbol{H}_{\mathrm{sca}}(\boldsymbol{r}) = \sum_{j=1,2}\left\{-i\frac{1}{Z}\frac{k^2}{\varepsilon_0\varepsilon_h}\boldsymbol{G}_{\mathrm{M}}(\boldsymbol{r}-\boldsymbol{r}_j).\boldsymbol{p}_j + k^2\boldsymbol{G}_{\mathrm{E}}(\boldsymbol{r}-\boldsymbol{r}_j).\boldsymbol{m}_j\right\}, \quad (4)$$

$\boldsymbol{G}_{\mathrm{E}}$ and $\boldsymbol{G}_{\mathrm{M}}$ being the free-space electric and magnetic dyadic Green's function respectively,

$$\boldsymbol{G}_{\mathrm{E}}(\boldsymbol{r}).\boldsymbol{p} = \left\{\left(1+\frac{i}{kr}-\frac{1}{k^2r^2}\right)\boldsymbol{p} + \left(-1-\frac{3i}{kr}+\frac{3}{k^2r^2}\right)(\boldsymbol{u}_r.\boldsymbol{p})\boldsymbol{u}_r\right\}g(r), \quad (5)$$

$$\boldsymbol{G}_{\mathrm{M}}(\boldsymbol{r}).\boldsymbol{p} = (\boldsymbol{u}_r\times\boldsymbol{p})\left(i-\frac{1}{kr}\right)g(r), \quad (6)$$

where $g(r)=e^{ikr}/4\pi r$ is the scalar Green's function and $\boldsymbol{u}_r$ is a unit vector along the direction of observation $\boldsymbol{r}$.

The electric and magnetic dipoles induced in each one of the particles are the result of the incident electric and magnetic fields (Supplementary Equations (1) and (2)) and the fields created by the interaction between both components of the dimer. They can be expressed as follows

$$\boldsymbol{p}_1 = \varepsilon_0\varepsilon_h\alpha_e\boldsymbol{E}_0(\boldsymbol{r}_1) + \alpha_e k^2\boldsymbol{G}_{\mathrm{E}}(\boldsymbol{r}_1-\boldsymbol{r}_2).\boldsymbol{p}_2 + i\varepsilon_0\varepsilon_h\alpha_e Zk^2\boldsymbol{G}_{\mathrm{M}}(\boldsymbol{r}_1-\boldsymbol{r}_2).\boldsymbol{m}_2, \quad (7)$$

$$\boldsymbol{m}_1 = \alpha_m\boldsymbol{H}_0(\boldsymbol{r}_1) - i\frac{\alpha_m}{Z}\frac{k^2}{\varepsilon_0\varepsilon_h}\boldsymbol{G}_{\mathrm{M}}(\boldsymbol{r}_1-\boldsymbol{r}_2).\boldsymbol{p}_2 + \alpha_m k^2\boldsymbol{G}_{\mathrm{E}}(\boldsymbol{r}_1-\boldsymbol{r}_2).\boldsymbol{m}_2, \quad (8)$$

$$\boldsymbol{p}_2 = \varepsilon_0\varepsilon_h\alpha_e\boldsymbol{E}_0(\boldsymbol{r}_2) + \alpha_e k^2\boldsymbol{G}_{\mathrm{E}}(\boldsymbol{r}_2-\boldsymbol{r}_1).\boldsymbol{p}_1 + i\varepsilon_0\varepsilon_h\alpha_e Zk^2\boldsymbol{G}_{\mathrm{M}}(\boldsymbol{r}_2-\boldsymbol{r}_1).\boldsymbol{m}_1, \quad (9)$$

$$\boldsymbol{m}_2 = \alpha_m\boldsymbol{H}_0(\boldsymbol{r}_2) - i\frac{\alpha_m}{Z}\frac{k^2}{\varepsilon_0\varepsilon_h}\boldsymbol{G}_{\mathrm{M}}(\boldsymbol{r}_2-\boldsymbol{r}_1).\boldsymbol{p}_1 + \alpha_m k^2\boldsymbol{G}_{\mathrm{E}}(\boldsymbol{r}_2-\boldsymbol{r}_1).\boldsymbol{m}_1, \quad (10)$$

where α_e and α_m are the electric and magnetic polarizabilities, which are related to the dipolar electric and magnetic scattering Mie coefficients, a_1 and b_1 respectively by means of the following expressions

$$\alpha_e = \frac{6\pi i a_1}{k^3} \text{ and } \alpha_m = \frac{6\pi i b_1}{k^3}. \quad (11)$$

The first terms on the right-hand side in Supplementary Equations (7) and (9) correspond to the electric dipoles in particles 1 and 2 respectively directly generated by the incident electric field. The second term in Supplementary Equation (7) (resp. Supplementary Equation (9)) corresponds to the electric dipole induced in particle 1 (resp. particle 2) by the electric dipole of particle 2 (resp. particle 1). The last term in Supplementary Equation (7) (resp. Supplementary Equation (9)) is the electric dipole induced in particle 1 (resp. particle 2) by the magnetic dipole of particle 2 (resp. particle 1). The physical explanation of Supplementary Equations (8) and (10) is similar. The second term in Supplementary Equation (8) (resp. Supplementary Equation (10)) corresponds to the magnetic dipole induced in particle 1 (resp. particle 2) by the electric dipole of particle 2 (resp. particle 1). The last term in Supplementary Equation (8) (resp. Supplementary Equation (10)) is the magnetic dipole induced in particle 1 (resp. particle 2) by the magnetic dipole of particle 2 (resp. particle 1).

The expressions of the dyadic Green's function for this particular case (particles located along the *Y*-axis) are given by

$$\boldsymbol{G}_{\mathrm{E}}(\boldsymbol{r}_1 - \boldsymbol{r}_2).\boldsymbol{e}_{\boldsymbol{x}} = \left\{\left(1 + \frac{i}{kD} - \frac{1}{k^2D^2}\right)\right\} g(D)\boldsymbol{e}_{\boldsymbol{x}} \equiv -g_{xx}\boldsymbol{e}_{\boldsymbol{x}}, \quad (12)$$

$$\boldsymbol{G}_{\mathrm{M}}(\boldsymbol{r}_1 - \boldsymbol{r}_2).\boldsymbol{e}_{\boldsymbol{x}} = -\left(i - \frac{1}{kD}\right) g(D)\boldsymbol{e}_{\mathbf{z}} \equiv g_{zx}\boldsymbol{e}_{\boldsymbol{z}}, \quad (13)$$

$$\boldsymbol{G}_{\mathrm{E}}(\boldsymbol{r}_1 - \boldsymbol{r}_2).\boldsymbol{e}_{\boldsymbol{z}} = \left\{\left(1 + \frac{i}{kD} - \frac{1}{k^2D^2}\right)\right\} g(D)\boldsymbol{e}_{\mathbf{z}} \equiv -g_{xx}\boldsymbol{e}_{\boldsymbol{z}}, \quad (14)$$

$$\boldsymbol{G}_{\mathrm{M}}(\boldsymbol{r}_1 - \boldsymbol{r}_2).\boldsymbol{e}_{\boldsymbol{z}} = \left(i - \frac{1}{kD}\right) g(D)\boldsymbol{e}_{\mathbf{x}} \equiv -g_{zx}\boldsymbol{e}_{\boldsymbol{x}}, \quad (15)$$

$$\boldsymbol{G}_{\mathrm{E}}(\boldsymbol{r}_1 - \boldsymbol{r}_2).\boldsymbol{e}_{\boldsymbol{y}} = \left\{\left(-\frac{2i}{kD} + \frac{2}{k^2D^2}\right)\right\} g(D)\boldsymbol{e}_{\mathbf{y}} \equiv g_{yy}\boldsymbol{e}_{\boldsymbol{y}}, \quad (16)$$

$$\boldsymbol{G}_{\mathrm{M}}(\boldsymbol{r}_1 - \boldsymbol{r}_2).\boldsymbol{e}_y = 0, \tag{17}$$

where $D = d+2R$ is the distance between the center of both spheres, *i.e.,* the distance between the two sets of dipoles. We also define a new dimensionless parameter, d_0, as the distance between the two particles, d, divided by the particle radius R. Similarly, we define the size parameter q as $2\pi R/\lambda$, which is also dimensionless.

Using Supplementary Equations (12-17), it is possible to obtain the expressions of the induced electric and magnetic dipoles in each particle. For the transverse polarization (electric field along the X-axis), they are given by

$$p_{1x} = \varepsilon_0\varepsilon_h\alpha_e E_0 - \alpha_e k^2 g_{xx} p_{2x} - i\varepsilon_0\varepsilon_h\alpha_e Z k^2 g_{zx}\, m_{2z}, \tag{18}$$

$$p_{1y} = \alpha_e k^2 g_{yy} p_{2y}, \tag{19}$$

$$p_{1z} = -\alpha_e k^2 g_{xx} p_{2z} + i\varepsilon_0\varepsilon_h\alpha_e Z k^2 g_{zx}\, m_{2x}, \tag{20}$$

$$m_{1x} = \frac{i\alpha_m}{Z}\frac{k^2}{\varepsilon_0\varepsilon_h} g_{zx} p_{2z} - \alpha_m k^2 g_{xx} m_{2x}, \tag{21}$$

$$m_{1y} = \alpha_m \frac{E_0}{Z} + \alpha_m k^2 g_{yy} m_{2y}, \tag{22}$$

$$m_{1z} = -\frac{i\alpha_m}{Z}\frac{k^2}{\varepsilon_0\varepsilon_h} g_{zx} p_{2x} - \alpha_m k^2 g_{xx} m_{2z}, \tag{23}$$

$$p_{2x} = \varepsilon_0\varepsilon_h\alpha_e E_0 - \alpha_e k^2 g_{xx} p_{1x} + i\varepsilon_0\varepsilon_h\alpha_e Z k^2 g_{zx}\, m_{1z}, \tag{24}$$

$$p_{2y} = \alpha_e k^2 g_{yy} p_{1y}, \tag{25}$$

$$p_{2z} = -\alpha_e k^2 g_{xx} p_{1z} - i\varepsilon_0\varepsilon_h\alpha_e Z k^2 g_{zx}\, m_{1x}, \tag{26}$$

$$m_{2x} = -\frac{i\alpha_m}{Z}\frac{k^2}{\varepsilon_0\varepsilon_h} g_{zx} p_{1z} - \alpha_m k^2 g_{xx} m_{1x}, \tag{27}$$

$$m_{2y} = \alpha_m \frac{E_0}{Z} + \alpha_m k^2 g_{yy} m_{1y}, \tag{28}$$

$$m_{2z} = \frac{i\alpha_m}{Z}\frac{k^2}{\varepsilon_0\varepsilon_h} g_{zx} p_{1x} - \alpha_m k^2 g_{xx} m_{1z}. \quad (29)$$

After solving the system of linear equations (Supplementary Equations (18-29)), the components of the resulting induced electric and magnetic dipoles are given by

$$p_{1y} = p_{2y} = m_{1x} = m_{2x} = p_{1z} = p_{2z} = 0, \quad (30)$$

$$p_{1x} = p_{2x} = \frac{\varepsilon_0\varepsilon_h \alpha_e E_0 (1 - \alpha_m g_{xx} k^2)}{t}, \quad (31)$$

$$m_{1y} = m_{2y} = \frac{\alpha_m E_0}{Z(1 - \alpha_m g_{yy} k^2)}, \quad (32)$$

$$m_{1z} = -m_{2z} = \frac{\alpha_e E_0 (-i\alpha_m g_{zx} k^2)}{Zt}, \quad (33)$$

with

$$t = 1 + \alpha_e g_{xx} k^2 - \alpha_m g_{xx} k^2 - \alpha_e \alpha_m {g_{xx}}^2 k^4 - \alpha_e \alpha_m {g_{zx}}^2 k^4. \quad (34)$$

From Supplementary Equation (30-33), we observe that the electric dipoles are excited in the direction of the incident electric field (*X*-axis) and the induced magnetic dipoles are perpendicular to the electric ones, i.e. they are along the *Y*- and *Z*-axis (see Supplementary Fig. 1b). It is worth noticing that the *Z*-component of the magnetic dipoles is not directly excited by the incoming external field. Indeed, the only component which is directly induced by the incident beam must be parallel to the magnetic incident field, that is, parallel to the *Y*-axis (see the first term in Supplementary Equations (8) and (10)). The *Z*-component of the magnetic dipole is thus appearing due to the coupling effect. It is null if g_{zx} is null, thus g_{zx} is an interaction term responsible for this extra magnetic component. Similarly, g_{xx} is also an interaction term. Indeed, when the particle distance increases, *D* tends to infinity, then g_{zx} and g_{xx} both tend to *0*, and Supplementary Equation (31) is simplified to $p_{1x} = p_{2x} = \varepsilon_0\varepsilon_h E_0 \alpha_e$, which is nothing but the first term in Supplementary Equations (7) and (9).

By introducing the previous expressions of the induced electric and magnetic dipoles into the equations of the far-field scattered electric fields, we get

$$\boldsymbol{E}_p^{\mathrm{ff}}(\boldsymbol{r}) \approx \frac{k^2 e^{ik|\boldsymbol{r}-\boldsymbol{r}_d|}}{4\pi\varepsilon_h\varepsilon_0|\boldsymbol{r}-\boldsymbol{r}_d|}[\boldsymbol{p}(\boldsymbol{r}_d) - (\boldsymbol{u}_r \cdot \boldsymbol{p}(\boldsymbol{r}_d))\boldsymbol{u}_r], \tag{35}$$

$$\boldsymbol{E}_m^{\mathrm{ff}}(\boldsymbol{r}) \approx -Z\frac{k^2 e^{ik|\boldsymbol{r}-\boldsymbol{r}_d|}}{4\pi|\boldsymbol{r}-\boldsymbol{r}_d|}[\boldsymbol{u}_r \times \boldsymbol{m}(\boldsymbol{r}_d)], \tag{36}$$

where $\boldsymbol{r}$ indicates the detector location, $\boldsymbol{r}_d$ the dipole location and $\boldsymbol{u}_r = \frac{\boldsymbol{r}-\boldsymbol{r}_d}{|\boldsymbol{r}-\boldsymbol{r}_d|}$.

$\boldsymbol{E}_{p1}^{\mathrm{ff}}$, $\boldsymbol{E}_{p2}^{\mathrm{ff}}$, $\boldsymbol{E}_{m1}^{\mathrm{ff}}$ and $\boldsymbol{E}_{m2}^{\mathrm{ff}}$ are the scattered electric fields in the far-field approximation created by the induced electric dipoles of the particles 1 and 2 and by the induced magnetic dipoles of both components of the dimer. For the right angle scattering configuration, θ=90°, $\boldsymbol{u}_r$will be parallel to $\boldsymbol{e_y}$ and the previous equations simplify into

$$\boldsymbol{E}_{p1}^{\mathrm{ff}}(90°) \approx \frac{k^2 e^{ik(r-\frac{D}{2})}}{4\pi\varepsilon_h\varepsilon_0 r} p_{1\mathrm{x}}\boldsymbol{e_x}, \tag{37}$$

$$\boldsymbol{E}_{p2}^{\mathrm{ff}}(90°) \approx \frac{k^2 e^{ik(r+\frac{D}{2})}}{4\pi\varepsilon_h\varepsilon_0 r} p_{2\mathrm{x}}\boldsymbol{e_x}, \tag{38}$$

$$\boldsymbol{E}_{m1}^{\mathrm{ff}}(90°) \approx -Z\frac{k^2 e^{ik(r-\frac{D}{2})}}{4\pi r} m_{1\mathrm{z}}\boldsymbol{e_x}, \tag{39}$$

$$\boldsymbol{E}_{m2}^{\mathrm{ff}}(90°) \approx -Z\frac{k^2 e^{ik(r+\frac{D}{2})}}{4\pi r} m_{2\mathrm{z}}\boldsymbol{e_x}. \tag{40}$$

The total electric field is given by

$$\boldsymbol{E}^{\mathrm{ff}} \approx \boldsymbol{E}_{p1}^{\mathrm{ff}} + \boldsymbol{E}_{p2}^{\mathrm{ff}} + \boldsymbol{E}_{m1}^{\mathrm{ff}} + \boldsymbol{E}_{m2}^{\mathrm{ff}}. \tag{41}$$

By means of Supplementary Equation (41), the scattered intensity can be obtained and consequently P_{L}(90°). In order to be consistent with the definition of the far-field

intensity computed by the Finite Element software[2], we compute the scattered intensity as

$$I_S = r^2 \left|\boldsymbol{E}^{\mathrm{ff}} \cdot \boldsymbol{e}_x\right|^2 \approx \left|\frac{k^2}{4\pi}\right|^2 \left|\frac{e^{-ik\frac{D}{2}}}{\varepsilon_h\varepsilon_0} p_{1\mathrm{x}} + \frac{e^{ik\frac{D}{2}}}{\varepsilon_h\varepsilon_0} p_{2\mathrm{x}} - Ze^{-ik\frac{D}{2}} m_{1\mathrm{z}} - Ze^{ik\frac{D}{2}} m_{2z}\right|^2. \quad (42)$$

A similar expression holds for I_P.

In Supplementary Fig. 2, we show the comparison of the scattered intensities I_S computed either with the Finite Element software or the dipole approximation from Supplementary Equations (37-42). Unless specified otherwise, all the computations performed in the Supplementary Information are made for R_1=R_2=R=9 mm and $\varepsilon = 15.7 + 0.3i$. The agreement between both methods is quite good in the dipolar region.

The null value for I_S at q=0.773 appears as the distance between the particles decreases, *i.e.* as their mutual interaction increases. This means that the interaction between both components of the dimer is responsible for this behaviour. To corroborate this assumption, in Supplementary Fig. 3 we have plotted I_S for smaller gaps ranging from d_0=2 to 0, while neglecting the interaction terms g_{zx} and g_{xx} in the Green's function computations. In that case, only the electric dipoles p_{1x} and p_{2x} are responsible for the values of I_S, as $m_{1z} = m_{2z} = 0$. As expected, I_S never reaches zero when d_0 is smaller than 2, moreover the behaviour of the curves completely differs from the ones shown in Supplementary Fig. 2. The fact that $I_S = 0$ when $d_0 = 2$ and $q \approx 0.8$ is simply due to the classical interaction of the two electric dipoles, which are at $D = \frac{q\,\lambda}{2\pi}(2 + d_0) \approx \frac{\lambda}{2}$ apart.

It is clear that the fact that we obtain a null value of I_S for small gaps appears as a consequence of the interaction between the particles. But such a null value is also only possible if the electric field presents an interference effect in the far field, when it is

created by the induced electric and magnetic dipoles in both particles even if they are less than $\lambda/2$ apart. Let us denote the following complex amplitudes associated to each particle

$$A_1 e^{ik\varphi_1} = \frac{p_{1x}}{\varepsilon_h \varepsilon_0} - Z m_{1z}, \tag{43}$$

$$A_2 e^{ik\varphi_2} = \frac{p_{2x}}{\varepsilon_h \varepsilon_0} - Z m_{2z}. \tag{44}$$

We can rewrite Supplementary Equation (42) as

$$I_s \approx \left|\frac{k^2}{4\pi}\right|^2 \left[|A_1|^2 + |A_1|^2 + 2\ A_1 A_2 \cos\big(k(\varphi_1 - \varphi_2 - D)\big)\right]. \tag{45}$$

The only way for I_S to be null is to have

$$A_1 = A_1 \qquad \text{and} \qquad k(\varphi_1 - \varphi_2 - D) = \pi + 2\,n\,\pi. \tag{46}$$

It corresponds to a situation where the electric field generated by the electric and magnetic dipoles in the first particle has the same amplitude than the one generated by the electric and magnetic dipoles in the second particle. The phase difference between the two electric fields is close to π but changes with the gap distance d_0.

We now define the ratio between the Z-component of the magnetic dipolar moment and the electric dipole moment as

$$T_z e^{ik\psi_z} = \frac{Z\varepsilon_0 \varepsilon_h m_{1z}}{p_{1x}} = \frac{-i\alpha_m g_{zx} k^2}{1 - \alpha_m g_{xx} k^2}\,. \tag{47}$$

It is worth noticing that this complex ratio depends on the magnetic polarizability α_m but not on the electric one α_e. Due to the relationship existing between p_{1x} and p_{2x} in Supplementary Equation (31), as well as between m_{1z} and m_{2z} in Supplementary Equation (33), we obtain

$$\frac{A_1}{A_2} e^{ik(\varphi_1 - \varphi_2)} = \frac{1 - T_z e^{ik\psi_z}}{1 + T_z e^{ik\psi_z}} \ . \tag{48}$$

The condition of complete destructive interference stated in Supplementary Equation (46) requires that

$$\frac{A_1}{A_2} e^{ik(\varphi_1 - \varphi_2 - D)} = \ -1 \ . \tag{49}$$

Combining Supplementary Equations (48) and (49) results in

$$T_z e^{ik\psi_z} = \frac{i}{\tan\left(k \frac{D}{2}\right)} \ . \tag{50}$$

To summarize, the only possibility for having $I_S = 0$ is to fulfill the two conditions simultaneously

$$k\psi_z = \frac{\pi}{2} + 2\, n\, \pi, \tag{51}$$

and

$$T_z \tan\left(k \frac{D}{2}\right) = 1 \ . \tag{52}$$

In Supplementary Fig. 4, we have plotted the phase $k\psi_z$ as well as $T_z \tan\left(k \frac{D}{2}\right)$ for several values of d_0. The values of d_0 (or equivalently D) which both satisfy the conditions of Supplementary Equations (51) and (52) are the only ones which enable to achieve a complete destructive interference. Unfortunately, these two equations are non-linear with respect to d_0. It is thus not possible to derive the exact value of the corresponding d_0 in close form nor to ensure that both conditions are satisfied with the same set of parameters. Nevertheless, the figures and a manual search (see Supplementary Fig. 5) indicate that the best match is provided when $d_0 \approx 1/3$ for a value of $q \approx 0.7728$ which is nothing but the switching frequency that has been determined experimentally.

The interference of the electric field created by the electric and magnetic dipoles in far field is responsible of the Fano shape of the resonance. In particular, its origin corresponds to the interference between the sharp dipolar magnetic resonance (the narrow channel) with the tail of the broader dipolar electric mode (which acts as the broad channel). In order to show more clearly this effect, in Supplementary Fig. 6 we represent the different terms contributing to the scattered intensity (Supplementary Equation (42)), as a function of the distance between the particles of the dimer. This equation can be rewritten as follows

$$I_S \propto r^2 \left|E_p + E_m\right|^2 = r^2 \left\{\left|E_p\right|^2 + |E_m|^2 + 2Re(E_p E_m^*)\right\}. \qquad (53)$$

Therefore, I_S is given by the coherent superposition of the fields created by the electric and magnetic dipoles induced in the dimer. The interferential term (last in Supplementary Equation (53)) corresponds to the interference between the electric field created by the electric and magnetic dipoles, $2Re(E_p E_m^*)$, where Re refers to the real part and * to the complex conjugate of a complex number. At the switching frequency, q = 0.773, as the distance between the particles decreases, the dipolar magnetic resonance increases, whilst the dipolar electric mode (background) exhibits a dip due to the transfer of energy from the dipolar electric mode to the magnetic one. Through the interferential term, a phase change from q = 0.72 to q = 0.77 is observed. It changes from positive (constructive interference) to negative (destructive interference) values. In Supplementary Fig. 7 we show these different contributions for d_0 = 2 and 1/3, corresponding to the cuts shown in Supplementary Fig. 6 (b, d, f) in green and pink respectively.

Additional transformation of Supplementary Equations (51) and (52) can be performed in order to understand the influence of the various configuration parameters on the

selection of the switching frequency. One can indeed note that $kD=q(2+d_0)$ and that the ratio can only be expressed in q and d_0

$$T_z e^{ik\psi_z} = \frac{6\,\pi b_1(q)\tilde{g}_{zx}(q,d_0)}{1-6\,\pi i\; b_1(q)\tilde{g}_{zx}(q,d_0)}\,, \tag{54}$$

where

$$\tilde{g}_{xx}(q,d_0) = -\left(1 + \frac{i}{q(2+d_0)} - \frac{1}{q^2(2+d_0)^2}\right)\frac{e^{iq(2+d_0)}}{4\pi q(2+d_0)}\,, \tag{55}$$

$$\tilde{g}_{zx}(q,d_0) = -\left(i - \frac{1}{q(2+d_0)}\right)\frac{e^{iq(2+d_0)}}{4\pi q(2+d_0)}\,. \tag{56}$$

Thus Supplementary Equations (51) and (52) are only depending on q, d_0 and the dielectric constant of the particle. In Supplementary Fig. 8, we plot the discrepancy between the left and right-hand-side of Supplementary Equation (52) when it is computed at the specific value of q which satisfies at best Supplementary Equation (51). The smaller the discrepancy is, the smaller I_S will be.

From Supplementary Fig. 8a, one can confirm the fact that the adequate distance d_0 is the same whatever the radius of the sphere is being used. The values $d_0 \approx 1/3$ and $q \approx 0.7728$ are thus corresponding to universal numbers for this specific value of permittivity. From Supplementary Fig. 8b, one can follow the influence of the permittivity on d_0. It seems that it is necessary to further part the spheres when the permittivity increases in order to observe a destructive interference effect in far-field.

This universal numbers are also of interest for the intensity in the longitudinal configuration as they correspond to the largest peak value of I_P, as shown in Supplementary Fig. 9. This is an ideal situation when building a switching device as this ensures the largest possible gap between the low state and the high state of the logical unit.

In the forward and backward direction, the distance between the particles and the detector is the same in Supplementary Equations (35-36). There is thus no extra phase difference induced which would generate supplementary interference effects. For the forward direction, the intensity corresponds to

$$I_s(0°) \approx \left|\frac{k^2}{2\pi}\right|^2 \left[\frac{p_{1x}}{\varepsilon_h\varepsilon_0} + Z\cos(\beta)\, m_{1z} + Z\sin(\beta)\, m_{1y}\right], \tag{57}$$

while for the backward direction, it is

$$I_s(180°) \approx \left|\frac{k^2}{2\pi}\right|^2 \left[\frac{p_{1x}}{\varepsilon_h\varepsilon_0} + Z\cos(\beta)\, m_{1z} - Z\sin(\beta)\, m_{1y}\right], \tag{58}$$

with tan(β) = 2r/D. When the receiver is in far-field, $\beta \rightarrow \pi/2$ and

$$I_s(0°) \rightarrow \left|\frac{k^2}{2\pi}\right|^2 \left[\frac{p_{1x}}{\varepsilon_h\varepsilon_0} + Zm_{1y}\right] \ , \tag{59}$$

$$I_s(180°) \rightarrow \left|\frac{k^2}{2\pi}\right|^2 \left[\frac{p_{1x}}{\varepsilon_h\varepsilon_0} + -Zm_{1y}\right]. \tag{60}$$

Let us now introduce a new ratio between the Y-component of the magnetic dipolar moment and the electric dipolar moment as

$$T_y e^{ik\psi_y} = \frac{Z\varepsilon_0\varepsilon_h m_{1y}}{p_{1x}}\,. \tag{61}$$

The intensity in the forward and backward direction can be rewritten as

$$I_s(0°) \approx \left|\frac{k^2}{2\pi}\right|^2 \left|\frac{p_{1x}}{\varepsilon_h\varepsilon_0}\right|^2 \left[1 + T_y^2 + 2T_y\cos\left(k\psi_y\right)\right]\,, \tag{62}$$

$$I_s(180°) \approx \left|\frac{k^2}{2\pi}\right|^2 \left|\frac{p_{1x}}{\varepsilon_h\varepsilon_0}\right|^2 \left[1 + T_y^2 - 2T_y\cos\left(k\psi_y\right)\right]\,. \tag{63}$$

The conditions for having a minimum intensity in forward or backward direction is at least to have

$$T_y = 1 \tag{64}$$

and

$$k\psi_y = \pi + 2n\,\pi \quad \text{for the forward direction,} \tag{65}$$

$$k\psi_y = 2n\,\pi \quad \text{for the backward direction.} \tag{66}$$

Unfortunately, Supplementary Equation (65) seems to be never fulfilled, while Supplementary Equation (66) is achievable, as shown in Supplementary Fig. 10. Nevertheless, Supplementary Figure 11 indicates that Supplementary Equations (64) and (65) are not satisfied simultaneously. The best agreement could be reached for $d_0=1/3$ but for q values corresponding to very small intensities values as indicated in Fig. 2. Thus, the forward and backward directions do not seem to be the most favourable ones in order to distinguish between I_S and I_P.

Supplementary Note 2

Sensitivity analysis. In order to assess the stability of the proposed configuration with respect to the various parameters, a sensitivity analysis has been performed by modifying the geometrical and electromagnetic values associated to this dimer configuration. The sensitivity to the distance d_0 between the two spheres has already been shown in Supplementary Fig. 9. The sensitivity with respect to the radius R of the sphere is provided in Supplementary Fig. 12, either plotted along the dimensionless parameter q or the wavelength. Supplementary Figure 12a clearly shows that the configuration can be directly translated to another wavelength scale as long as the ratio between R and λ is maintained constant. As well, Supplementary Figure 12b shows that

for a given wavelength, there is a certain range of acceptable R values for which the switching effect is still activated.

A similar analysis is performed this time with respect to the permittivity value of the spheres as shown in Supplementary Fig. 13, showing again that the system is stable in the considered permittivity range.

We have also played with the incidence and reception angle directions. In Supplementary Fig. 14, the simulations have been performed for several detection directions close to 90°. In Supplementary Fig. 15, the spheres have been rotated either along the Z axis (which simply corresponds to a change in the incident polarizations directions), or along the X axis (which corresponds to a more complicated situation). Nevertheless, the switching effect is still visible. This sensitivity analysis thus clearly indicates that there is a large tolerance with respect to the variations of the geometrical and electromagnetic parameters, which renders the realization process less arduous.

Supplementary Methods

Far-field computation and calibration. The electric field in the far field can be computed using the Stratton-Chu formula[3]

$$\boldsymbol{E}^{\mathrm{ff}}(r) = \frac{-ike^{ikr}}{4\pi r}\frac{\boldsymbol{r}}{r}\times\iint\left[\boldsymbol{n}\times\boldsymbol{E}(\boldsymbol{r}') - Z\frac{\boldsymbol{r}}{r}\times\left(\boldsymbol{n}\times\boldsymbol{H}(\boldsymbol{r}')\right)\right]e^{-ik\boldsymbol{r}\cdot\boldsymbol{r}'}dS, \qquad (67)$$

where $\boldsymbol{n}$ is the normal at the surface S enclosing the radiating scattering sources. In the finite element method employed here[2], the provided output in far-field corresponds to the scattering amplitude

$$\boldsymbol{S}^{\mathrm{ff}}_{\mathrm{FEM}}(\vec{r}) = re^{-ikr}\boldsymbol{E}^{\mathrm{ff}}(\boldsymbol{r}). \qquad (68)$$

Thus, the intensity which is being considered herein corresponds to the intensity associated to this scattering amplitude, as already stated in Supplementary Equation (44), when measured along the polarization $\boldsymbol{v}$,

$$I^{\mathrm{FEM}} = \left|\boldsymbol{S}_{\mathrm{FEM}}^{\mathrm{ff}}(\boldsymbol{r}) \cdot \boldsymbol{v}\right|^2 = r^2\left|\boldsymbol{E}^{\mathrm{ff}}(\boldsymbol{r}) \cdot \boldsymbol{v}\right|^2. \qquad (69)$$

In order to be consistent, the intensity extracted from the measurement is computed in a similar way

$$I^{\mathrm{meas}} = L^2|C\,\boldsymbol{E}^{\mathrm{meas}}(\boldsymbol{r}) \cdot \boldsymbol{v}|^2, \qquad (70)$$

where *L* corresponds to the distance between the centre of the target and the receiver (here $L \approx 1.8$ m), and *C* is a complex calibration coefficient. Indeed, to obtain quantitative comparisons, all the fields have been simulated assuming an incident plane wave with unitary amplitude and null phase at the center of the target. The calibration coefficient *C* has been determined thanks to auxiliary measurements, i.e., the field measured with a single centered metallic sphere, which has been compared to the field simulated with Mie formulation[4].

Supplementary references

1. Albella, **P.** *et al.*, Low-Loss Electric and Magnetic Field-Enhanced Spectroscopy with Subwavelength Silicon Dimers. *J. Phys. Chem.* **117**, 13573−13584 (2013).

2. COMSOL Multiphysics 5.0; Comsol Inc.: Burlington, MA, 2015.

3. Chew, W.C., *Waves and Fields in Inhomogeneous Media.* (Van Nolstrand Reinhold, 1990)

4. Geffrin, J-M., Eyraud, C., Litman, A. & P. Sabouroux, Optimization of a bistatic microwave scattering measurement setup: From high to low scattering targets, *Radio Science* **44**, RS2007 (2009)
